# International Publication Trends in Low Back Pain Research: A Bibliometric and Visualization Analysis

**DOI:** 10.3389/fpubh.2022.746591

**Published:** 2022-03-02

**Authors:** Fan Huang, Beisi Zheng, Cunshu Wu, Siyi Zhao, Yuanyue Xu, Ziyuan Li, Chuyu Huang, Zhiyong Fan, Shan Wu

**Affiliations:** ^1^The Second School of Clinical Medicine, Guangzhou University of Chinese Medicine, Guangzhou, China; ^2^Acupuncture, Moxibustion, and Rehabilitation Clinical Medical College, Guangzhou University of Chinese Medicine, Guangzhou, China

**Keywords:** low back pain, bibliometric analysis, visualization, publications, trend

## Abstract

**Background:**

Although there is a growing research base on low back pain, the bibliometric literature related to it is deficient. The aim of this study was to conduct a bibliometric and visualization analysis of low back pain and to provide a broad view of the current trends in LBP research and a potential guide in this discipline.

**Methods:**

The authors searched the Web of Science to extract publications regarding low back pain, and found a total of 12,249 publications during a period of 22 years, among which 12,242 were eligible. We classified and analyzed publications such as total citations, average citations per item, H-index, research types, countries/regions, institutions, and journals using standard bibliometric indicators. Bibliometric approaches (VOSviewer1.6.13 and CiteSpace 5.8.3) were also available for gathering information and explore the trends of research.

**Results:**

Conspicuously, over the past 22 years, an increasing number of scholars have specialized in the research of LBP, exerting the boom in articles. The largest number of document type was that of articles. Under modern conditions, regional distinction existed in the research of low back pain and developed countries preceded others. Research individuals and institutions were preoccupied by respective aspects. Visualization analysis provided objective information for potential collaborators and cooperative institutions. Furthermore, most burst keywords varied during different periods.

**Conclusions:**

The map of research on LBP obtained by our analysis is expected to help researchers to efficiently and effectively explore LBP.

## Introduction

Under the global background of population aging, low back pain (LBP) is the leading cause of both reduction in working hours and disability (1, 2). LBP is a common condition across populations as the prevalence of LBP gradually augments by 11.4% each year, and up to 60–80% of the population in the world encounter this problem (3). Recent studies have described that LBP will cause 40% of nonattendance at work and reduce the affected individuals' productivity (4, 5). In America, economic loss due to reduction of productivity by LBP amounted to $100 billion in 2006 (6–8). To the best of our knowledge, only a small proportion of people has well understood pathological causes, such as vertebral fracture, malignancy, and infection (9, 10). Except for pathological causes of LBP, nonspecific LBP is ascribed to lifestyle factors, obesity, occupations that require sitting, and depression (11). The United States Institute of Medicine report Relieving Pain in America urges recognition of the complex, multidimensional nature of pain (biological, psychological, and social domains contribute to each individual's unique pain experience) (12).

The number of literature and the amount of funds on LBP are growing with increasing attention to LBP (8). The concentration of research has contributed to alleviation of long-term suffering from LBP and reduction in medical economic burden. Additionally, more achievements in scientific research are published in various journals in the form of articles (13). Bibliometrics is the application of qualitative and quantitative statistical analyses to a published study, as it can accurately characterize the current status of LBP research, including but not limited to the range of research topics, evolution of research topics, latest topics, and publishing trends (14–17). Although some bibliometrics studies have made attempts on LBP, there are still some shortcomings on the quality and timeliness of literature. Prior studies have failed to report the overall research status of LBP in the world. Researchers need to devote a significant amount of time to reading and identifying relevant studies in related fields because of long research interval, large amount of data, uneven quality of scientific research articles, presence of unnecessary duplications, and differences. Therefore, it is particularly urgent to sort out important, effective, and meaningful information from large databases in order to guide scientific research, and promote the proper prevention, control, diagnosis, and treatment of LBP.

Accordingly, bibliometric approaches were used to explore the production and development of current research. Our study seeks to provide a broad view of current trends in LBP and serve as a valuable reference and guidance for clinicians.

## Methods

### Data Sources and Search Strategy

We chose Web of Science as the data source to identify and extract relevant publications. In order to cover as many target documents as possible, we selected terms that might be used by most scientific publications to construct a search strategy. A search strategy is shown in Table 1. The strategy was searched in January 2022, yielding a total of 12,249 hits that were downloaded into Endnote X7. Moreover, we screened titles and abstracts in relation to inclusion and exclusion criteria. The inclusion criteria were as follow: (1) published in English, (2) published between 2000 and January 2022, (3) only articles and reviews, and (4) content of the articles was primarily focused on LBP. Studies that met the following were excluded: (1) publication type was animal experiment, (2) repeated publications, and (3) non-article-type documents (e.g., book review, notification, editorial materials, meeting abstracts, proceedings papers, letters, news items, and corrections).

**Table 1:** Electronic search strategy.

**Electronic database**
Web of Science (http://isiknowledge.com)
**Search strategy for the Web of Science database**
Search 1: TI = (“Low Back Pain”, “Back Pain, Low”, “Back Pains, Low”, “Low Back Pains”, “Pain, Low Back”, “Pains, Low Back”, “Lumbago”, “Lower Back Pain”, “Back Pain, Lower”, “Back Pains, Lower”, “Lower Back Pains”, “Pain, Lower Back”, “Pains, Lower Back”, “Low Back Ache”, “Ache, Low Back”, “Aches, Low Back”, “Back Ache, Low”, “Back Aches, Low”, “Low Back Aches”, “Low Backache”, “Backache, Low”, “Backaches, Low”, “Low Backaches”, “Low Back Pain, Postural”, “Postural Low Back Pain”, “Low Back Pain Posterior Compartment”, “Low Back Pain, Recurrent”, “Recurrent Low Back Pain”, “Low Back Pain, Mechanical”, “Mechanical Low Back Pain”)(combined by OR operator). Search 2: T2 = (“article”, “review”)(combined by OR operator). Final Search: (Search 1 AND Search 2)

### Statistical Analysis

We used Endnote X7 (https://endnote.com/) to exclude publications and further screen after reading the information. Total publications, research types, research orientations, research organization, author's contribution, and journal and publications situation (total citation frequency, average citation per item, and H-index) were analyzed statistically with the HistCite software to mitigate disagreements. We used VOSviewer1.6.13 to perform a bibliometric analysis and establish visualization knowledge maps. Figures were processed with the Adobe Illustrator CS5 software (Adobe Corporation). A bibliometric analysis platform (https://bibliometric.com) was used to analyze international collaborations between countries/regions. Furthermore, we used CiteSpace 5.8.3 to analyze strongest citation bursts. In order to analyze year related to publication time and impact factor (IF) related to journal, we used the IBM SPSS Statistics software 22.0 to conduct Pearson's correlation analysis.

## Results

### Type of Documents

**Figure 1:**
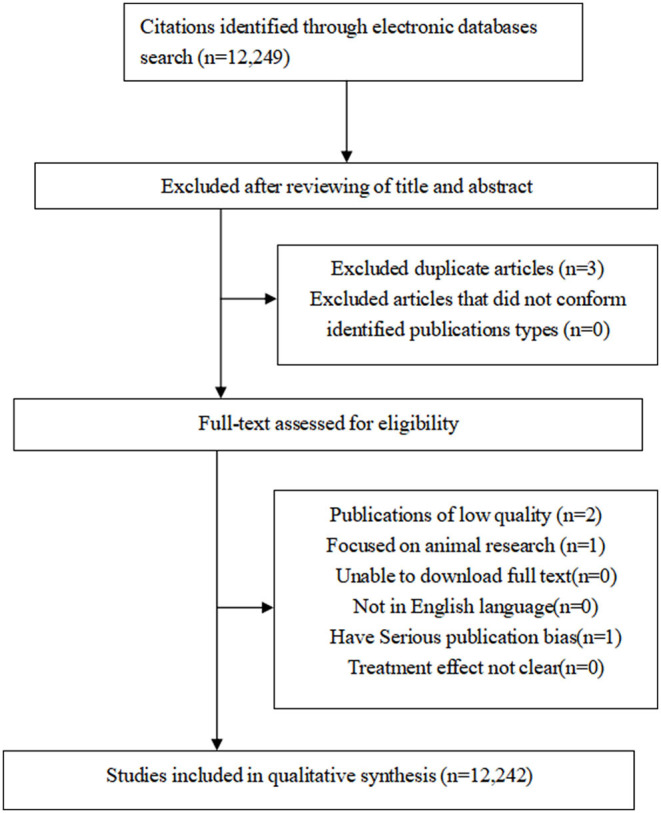
Flow chart of selection processes for eligible studies.

Fourteen document types were found in 12,242 publications from 2000 to 2022 (Figure 1). Articles ranked first, accounting for 67.72% of total frequency in document type, followed by meeting abstracts (11.13%), reviews (9.43%), editorial materials (5.09%), letters (5%), corrections (1.23%), proceedings articles (1.19%), early access (0.84%), new items (0.34%), reprints (0.02%), retracted publications (0.02%), retractions (0.02%), book chapters (0.02%), and book reviews (0.01%).

### Chronological Growth

**Figure 2:**
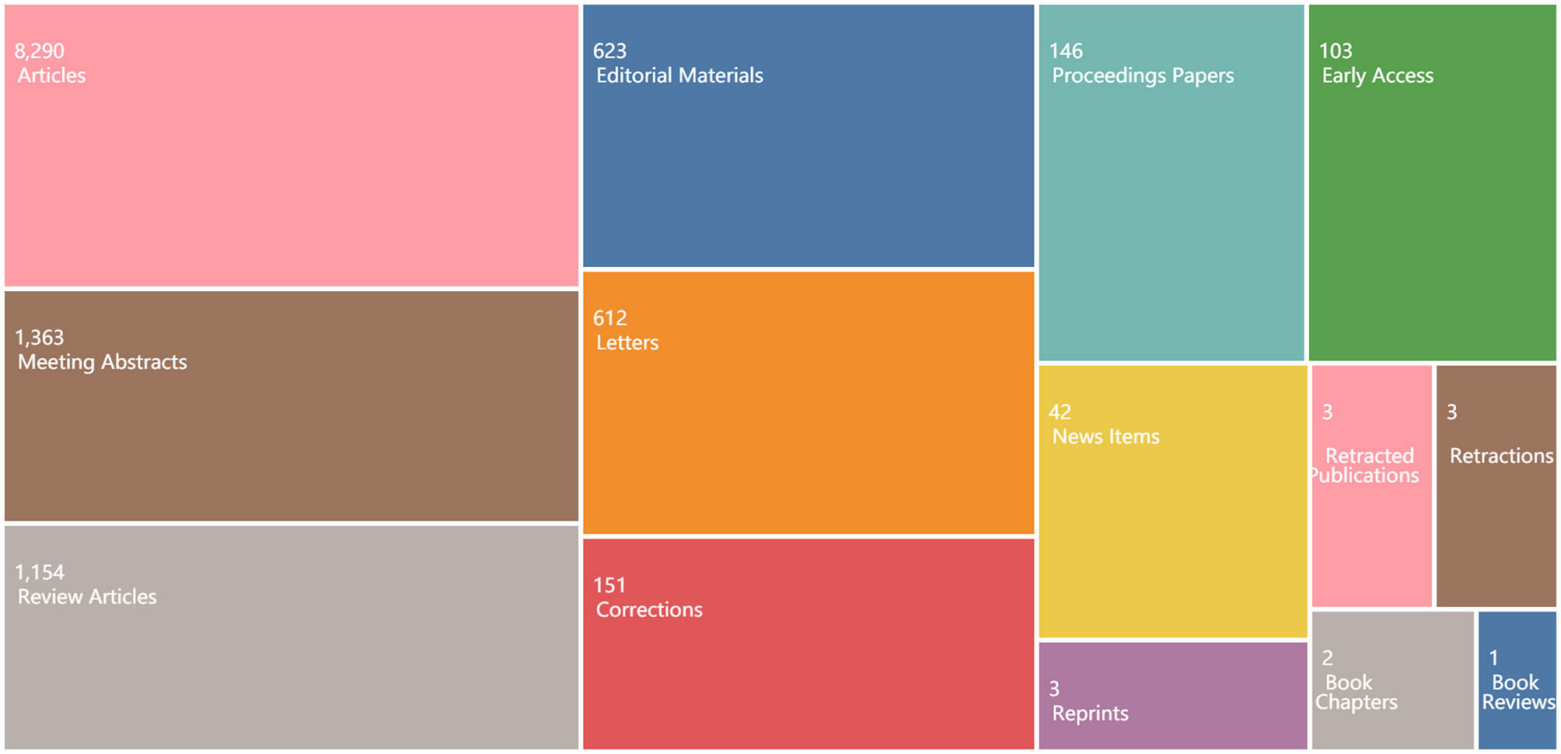
Treemap chart of document types.

**Figure 3:**
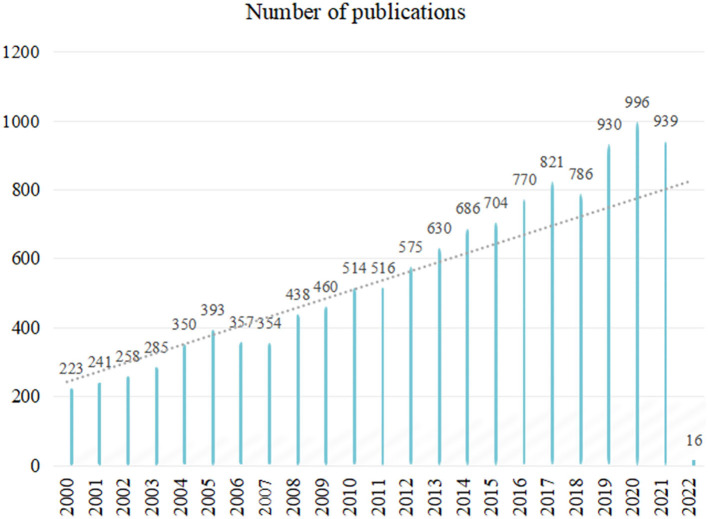
Number of publications per year and growth trend for low back pain (LBP).

A total number of 12,249 records were retrieved. In accordance with the inclusion and exclusion criteria, our study eventually included and evaluated further 12,242 articles with the flow diagram of data collection process (Figure 2). As shown in Figure 3, the publication outputs went through a period of fluctuation between 2000 and 2007. Conversely, the number of published articles increased rapidly after 2008. In order to clearly predict the trend of LBP studies, Pearson's correlation analysis was performed and revealed that the publication outputs are moderately correlated to the year of publication (*r* = 0.682, *p* = 0 < 0.001).

### Distribution of Output and Impact of Most Prolific Country

**Figure 4:**
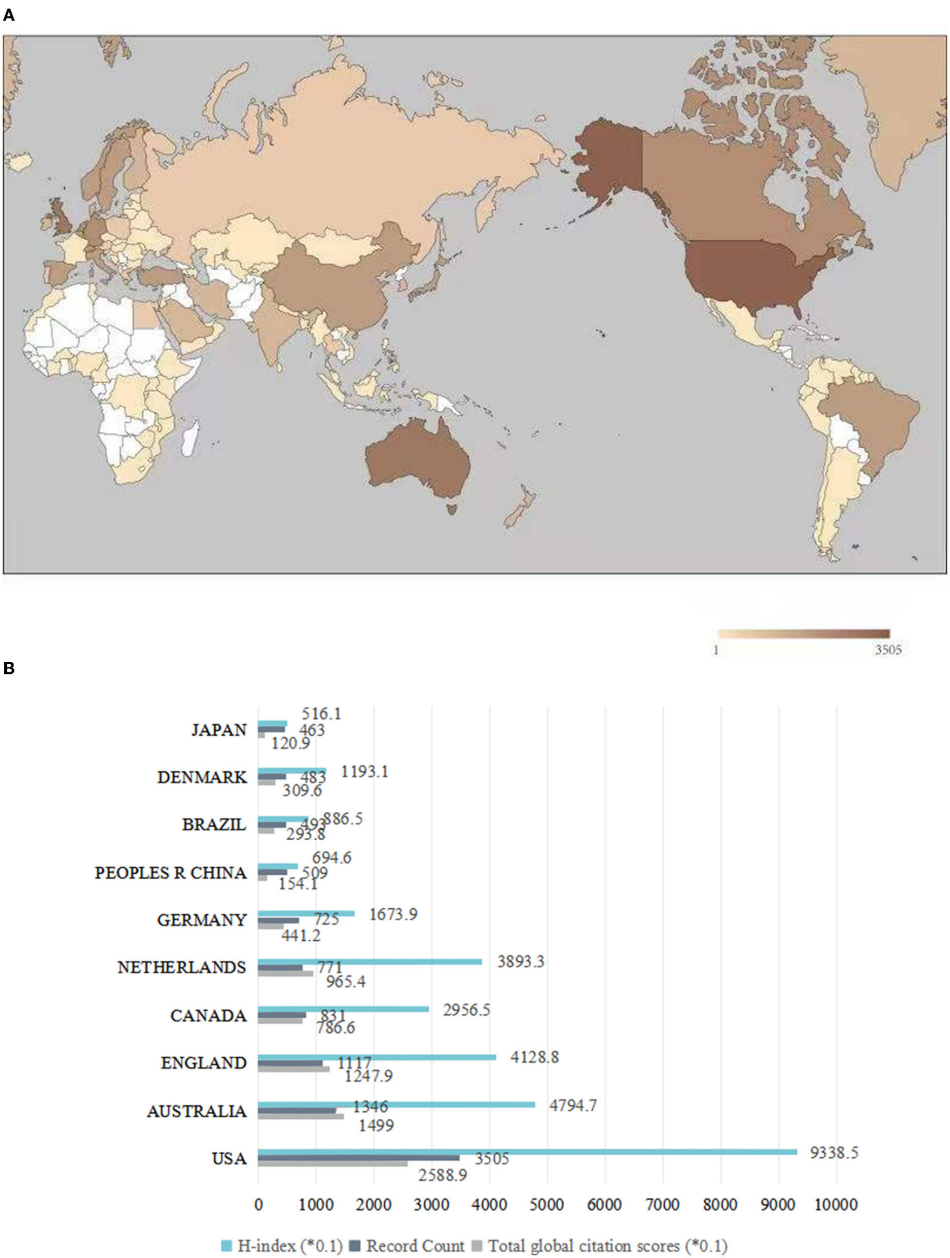
Articles in the world related to LBP. **(A)** Distribution of LBP publications. **(B)** Number of publications, total local citation score (number of citations of country or region publications in Web of Science), and H-index among top 10 productive countries.

The 12,242 articles were published in journals of 117 countries/regions participating in LBP research (Figure 4A). Among them, the United States published the most articles (28.6%), followed by Australia (10.99%), England (9.12%), Canada (6.79%), Netherlands (6.3%), Germany (5.92%), China (4.16%), Brazil (4.03%), Denmark (3.95%), and Japan (3.78%) (Figure 4B). This demonstrated that there are regional differences in the degree of research on LBP. The most productive countries (regions) were the United States. Asian countries participated in the publication of articles with a relatively low number. The United States plays a leading role in the research on LBP, with the highest total local citation score and H-index.

### Institutions and Authors

**Figure 5:**
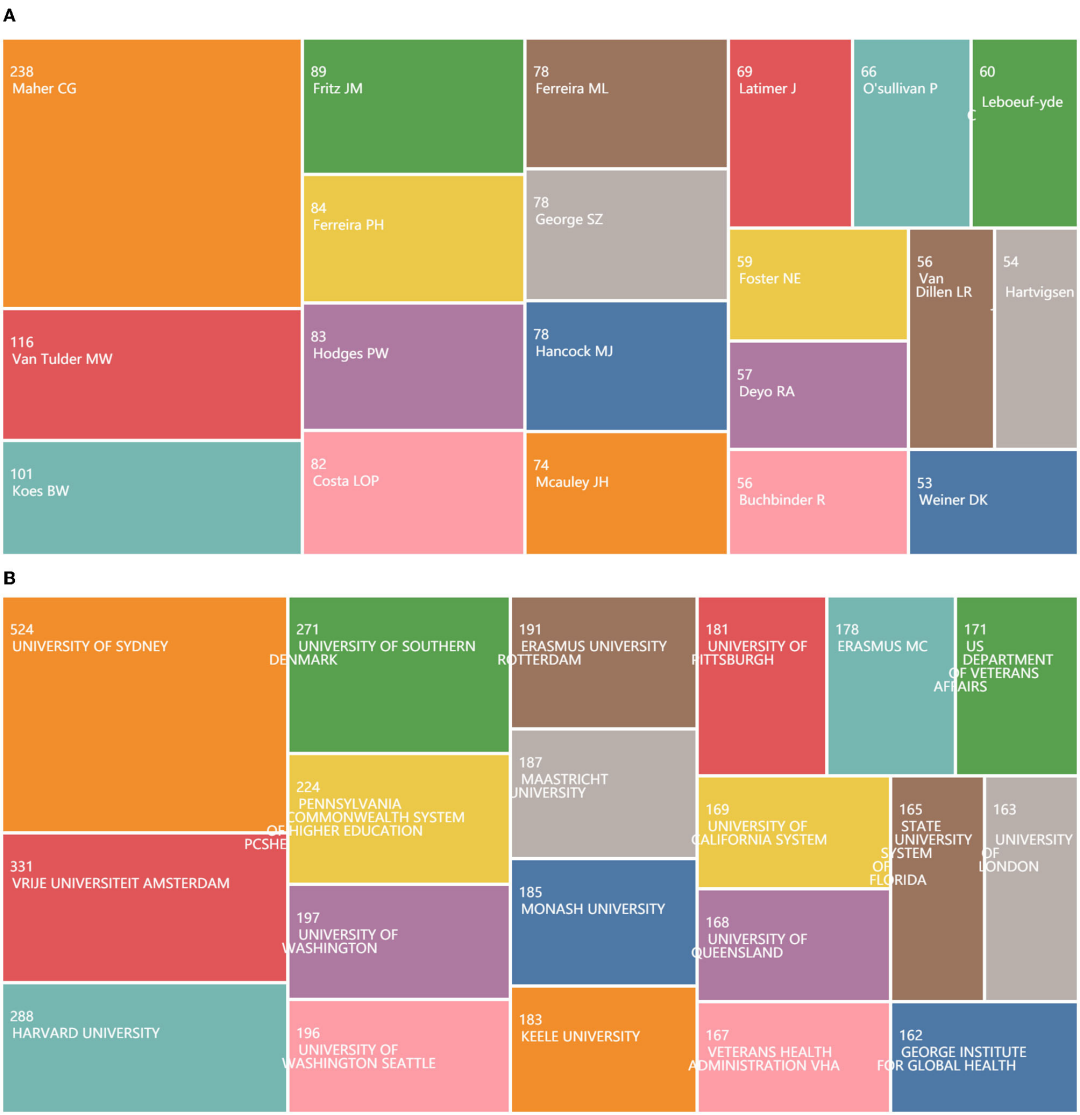
**(A)** Treemap chart of authors. **(B)** Treemap chart of institutions.

The total number of authors included in the 12,242 articles related to LBP was 30,650, with an average of 2.5 authors per article. Figure 5A presents the 20 most productive authors in LBP research. In addition, Figure 5B presents the 20 most productive among a total of 9,624 institutions.

### Highly Cited Articles

Out of 10 most cited publications on LBP, 6 were articles and 4 were reviews. Table 2 shows a list of the most cited empirical articles on LBP. The two most cited publications were clinical practice guidelines, which provided recommendations on the diagnosis and management of LBP. Also included among the 10 most cited empirical articles were systematic reviews validating the prevalence of LBP.

**Table 2:** Top 10 cited articles related to low back pain (LBP).

**Rank**	**Title**	**H-index**
1	Diagnosis and treatment of low back pain: A joint clinical practice guideline from the American college of physicians and the American pain society	1,856
2	Chapter 4—European guidelines for the management of chronic nonspecific low back pain	1,457
3	A systematic review of the global prevalence of low back pain	1,271
4	The global burden of low back pain: estimates from the Global Burden of Disease 2010 study	1,242
5	Primary care—Low back pain	1,182
6	Interpreting change scores for pain and functional status in low back pain—Toward international consensus regarding minimal important change	1,175
7	A systematic review of low back pain cost of illness studies in the United States and internationally	1,159
8	Lumbar disc disorders and low-back pain: Socioeconomic factors and consequences	954
9	The Epidemiology of low back pain	910
10	What low back pain is and why we need to pay attention	906

### Areas of Research

**Figure 6:**
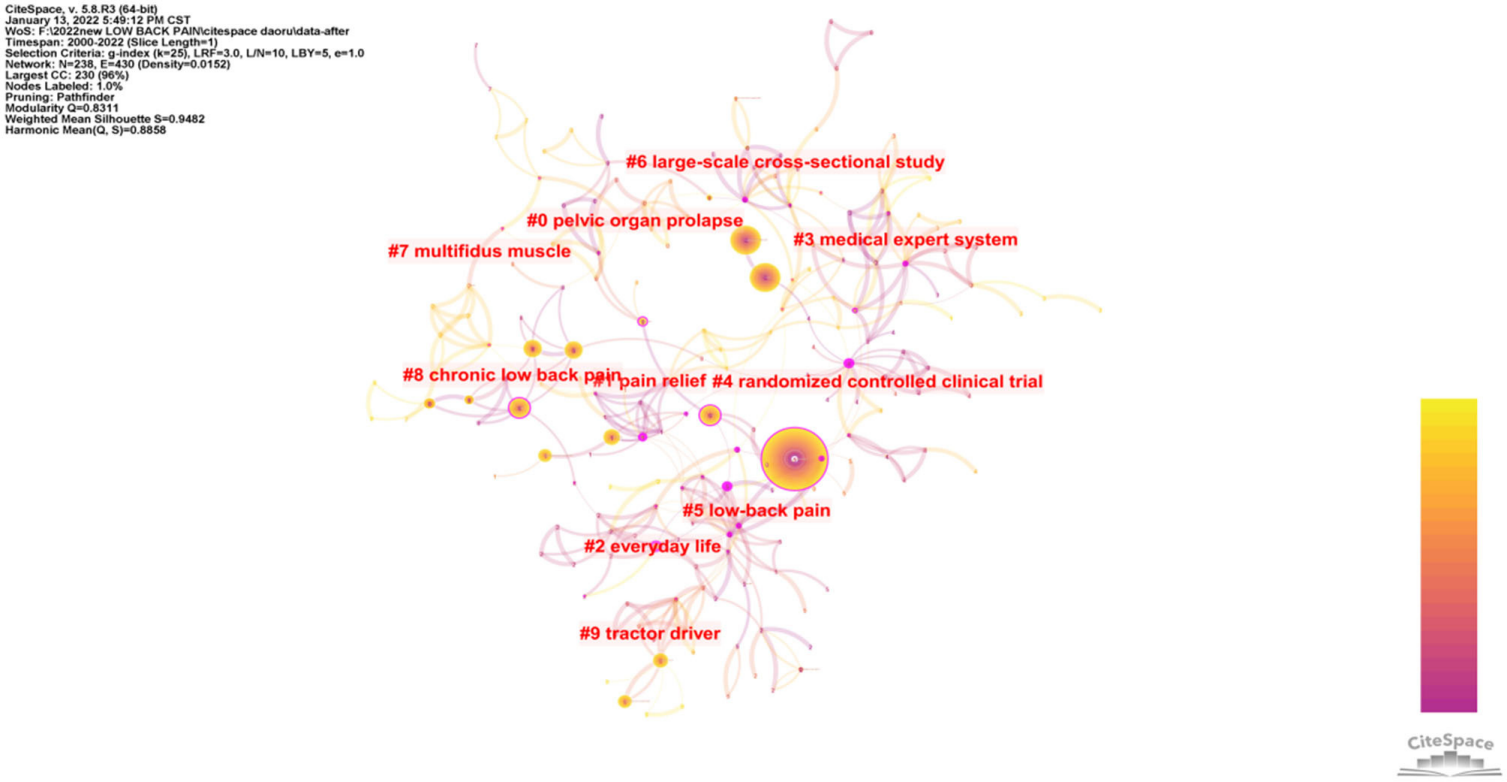
Cluster of the areas of research.

The articles covered 85 areas of research. There are 10 clusters in the main field of LBP (Figure 6). The 10 clusters cover pelvic organ prolapse, pain relief, everyday life, medical expert system, randomized controlled clinical trial, low back pain, large-scale cross-sectional study, multifidus muscle, chronic LBP, and tractor driver.

### Pattern of Collaboration

**Figure 7:**
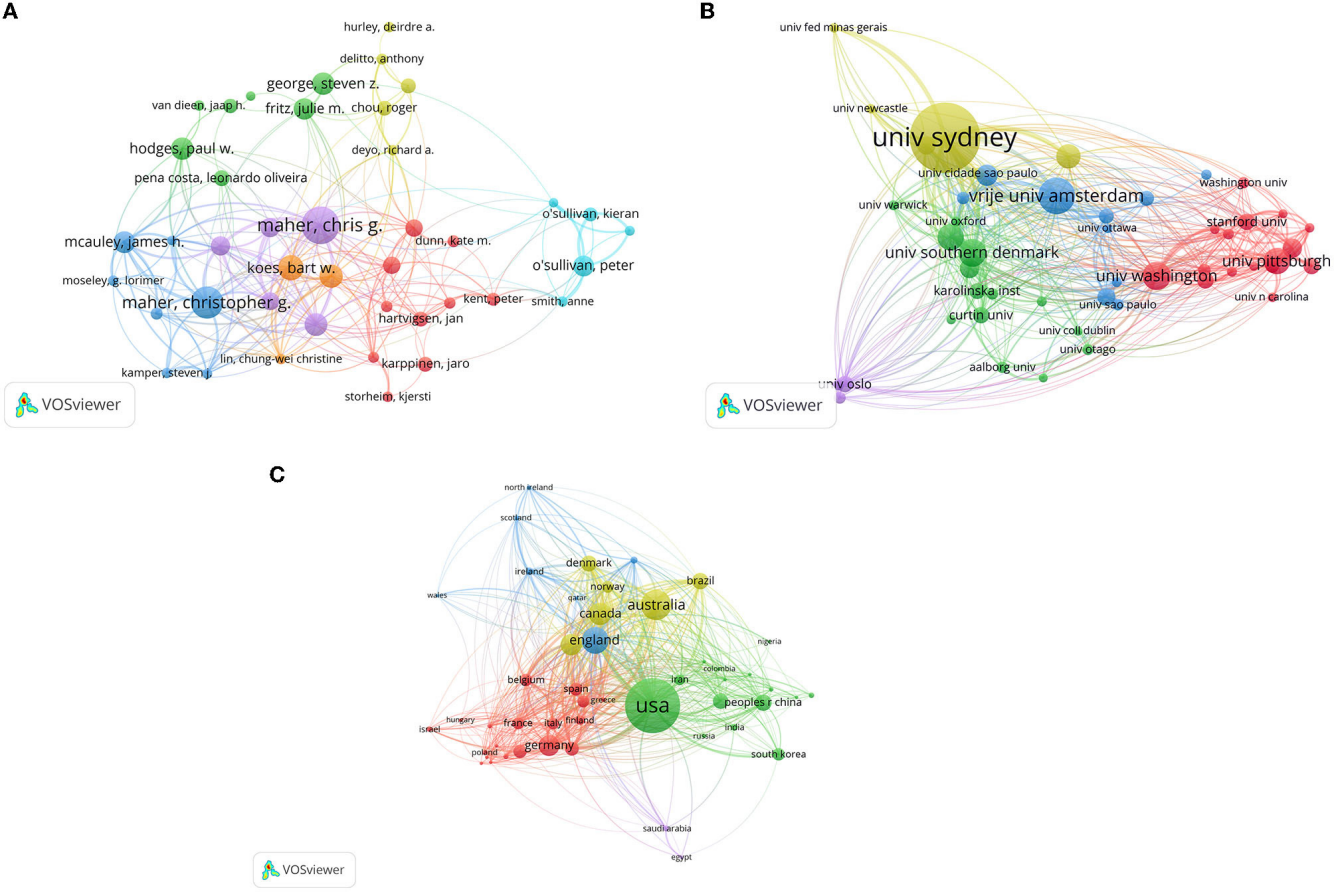
Collaboration of authors, institutions, and countries/regions for LBP research. The thickness of the link between any two authors, institutions, and countries/regions is indicative of the extent of co-authorship, and the colors of the circles indicate groups of authors, institutions, and countries/regions with a high degree of collaboration. **(A)** Network map of authors. **(B)** Network map of institutions. **(C)** International collaboration between countries/regions.

The network map showed some dominant teams (Figure 7A). Maher, Christopher G.; Mcauley, James H.; Moseley, G.; Lorimer, Kamper; Steven, J.; and Lin, Chung-wei Christine cooperated closely. In the collaboration of institutions (Figure 7B), 9,624 institutions were involved in LBP research. University of Sydney has the strongest partnerships with Macquarie University, University of Newcastle, University of Fed Minas Gerais and University of Cidade Saulo., revealing high academic influence and board collaboration in LBP research. In the collaboration of countries/regions (Figure 7C), the United States was the most engaged in international cooperation, followed by Australia, Netherlands, Canada, and United Kingdom. The United States and Brazil, as well as Belgium and Netherlands, had a cohesive collaboration. Visualization analysis provides objective information for researchers to identify new perspectives on potential collaborators and cooperative institutions, which promote immediate cooperation.

### Keywords

**Figure 8:**
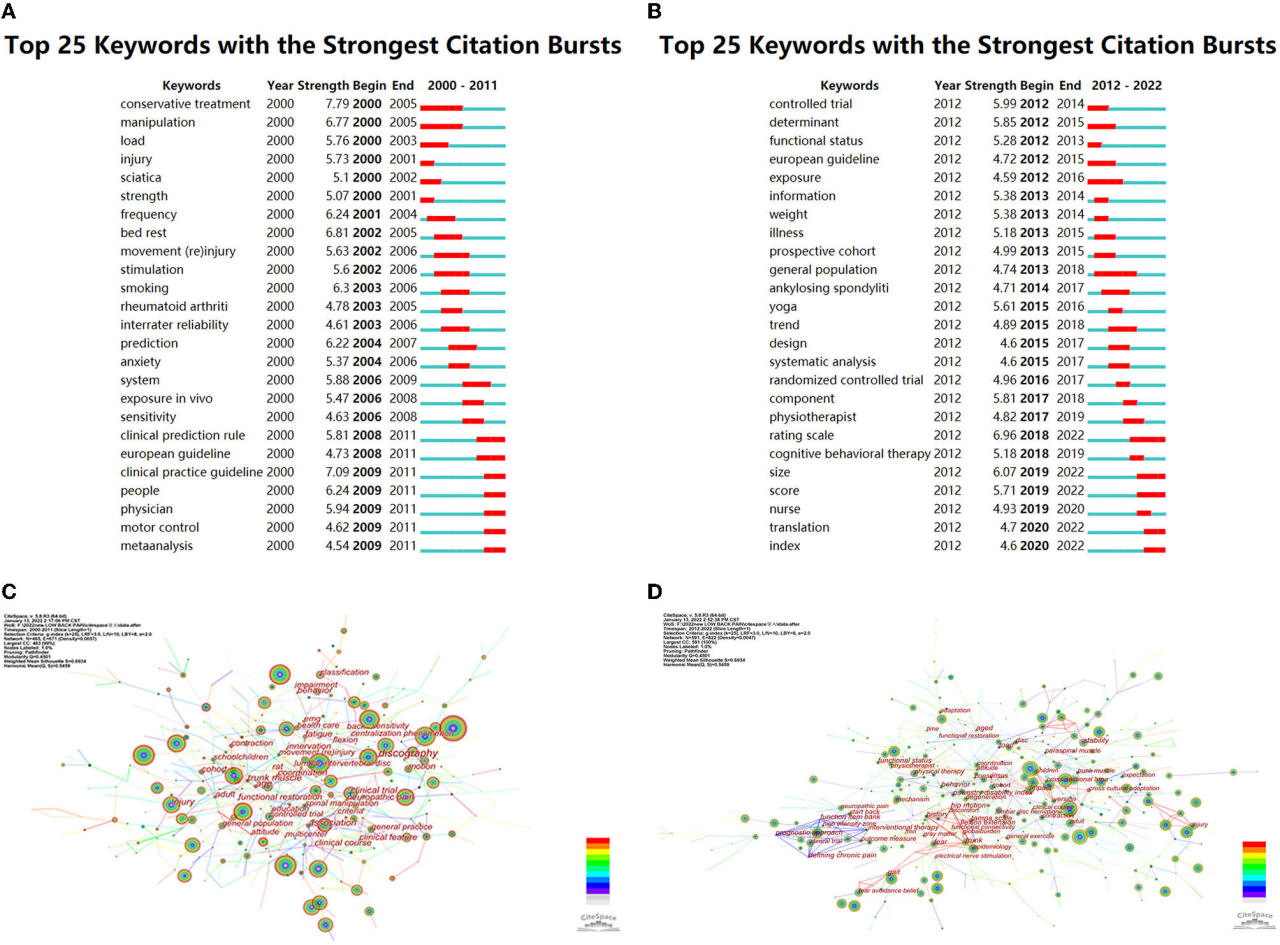
Keywords for LBP research. **(A)** Top 25 keywords with strongest citation bursts of publications on LBP research from 2000 to 2011. **(B)** Top 25 keywords with strongest citation bursts from 2012 to 2022. **(C)** Network map of keywords from 2000 to 2011. **(D)** Network map of keywords from 2012 to 2022.

The top 25 keywords with strongest occurrence burst are shown in Figures 8A, B. Occurrence burst, which indicates a steep increase in occurrence over a period of time, represents changes in frontier topics and dynamics in a research field. In the following years, the occurrence of 25 keywords declined, which demonstrated that researchers contiguously strengthen research and infuse new blood into the development of LBP research. From the network map of keywords (Figures 8C, D), the control trial of LBP studies had increased in last 11 years, becoming a hot topic for research. The keywords disability, management, and prevalence had the highest occurrence, revealing that etiology, treatment, effect, and prognosis have become the leading direction of LBP research, while from 2000 to 2011, disability, management, and primary care had the highest occurrence.

## Discussion

### Summary of the Research

The published articles over the last 22 years demonstrated that greater emphasis is now placed on LBP research. Albeit with concerted efforts from all countries/regions, developed countries such as the United States and United Kingdom played a crucial role during this period. A startling discovery is that universities and other teaching institutions become the forefront of academic research. Journals related to etiology and treatment of LBP stand out. Furthermore, the most burst keywords were primary-care, lumbar spine and disability in the field of LBP, among of which showed the research focus of LBP.

Clinically, a certain disease roughly undergoes some research process that includes pathogenesis, pathology, clinical manifestation, diagnosis, treatment, and prevention. We used VOSviewer and CiteSpace to visualize the frequency of occurrence and trend of keywords in the wake of time, and we found that the current research is focused on the pathology, classification, and diagnosis of LBP. By keyword analysis, the research hotspot was biased toward the treatment method from 2000 to 2011, while research paid more attention to evidence-based medicine from 2012 to 2022. This era of groundbreaking scientific developments in high-resolution, high-throughput technologies is allowing the cost-effective collection and analysis of huge, disparate sets of data on individual health. Evidence-based medicine could summarize the diagnosis and treatment of LBP and provide guidance for clinical practice (18). LBP is a complicated symptom that has many factors. The factors could be roughly divided into physical agents (such as prolonged standing and lifting heavy weights) unhealthy lifestyle (such as smoking and obesity), and psychological factors (such as distress and expectations that pain indicates bodily harm or injury) (19, 20). Clinically, when the pathological cause of LBP cannot be identified, the pain will be classified as nonspecific (1). Currently, there is no well-accepted single classification, since the taxonomy of LBP is underdeveloped (21). Some studies indicated that there is a classification system for LBP, such as mechanical or neuropathic in clinical practice (22), which was consistent with our analysis of subject categories. In the management of LBP, musculoskeletal system and nervous system hold a great potential (23). Despite intensive research focus on LBP, definitive diagnostic methods are largely unavailable, and standard terminology is not yet broadly adopted (22). Therefore, it is necessary to develop consensus diagnostic methods and standard terminology for the application of LBP as tools to guide clinicians toward a better mastery of clinical decisions, focusing on accurate diagnosis and detection of red flags and avoiding the establishment of false-positive or false-negative diagnoses (24). Treatment of LBP is also a hot topic of research. On the basis of the maps of our analysis, physiotherapy, drugs, surgical treatment, and complementary and alternative medicine were the vital research contents of treatment, whereas over time, the research on drug and surgical treatment decreased gradually. Some evidence showed that there is wide acceptance that the management of LBP should begin in primary care (25). With a strong growth in research on different therapy, developing comprehensive and multilevel guidelines to provide specific recommendations to fill the gaps in research is likely to become increasingly important in the world (26, 27). At present, the guidelines will be updated within 3–5 years after publication if new evidence alters the recommendations (28). The current guidelines urgently need more clinical trials to conduct reasonable research on the diagnosis and treatment of LBP (29).

### Current Studies of Bibliometric Analysis on LBP

Over the last years, there appeared two kinds of bibliometric analysis studies on LBP. One provided insight into the trends of development in the application of specific treatments for LBP, such as acupuncture and exercise, and the other concentrated on evaluation of the research situation and captured subsequent developmental dynamics regarding nonspecific LBP and occupational LBP (13, 30–33). Compared with previous studies, we generated a comprehensive strategy and did not limit the document types of LBP or did not present specific treatment for LBP. Such a bibliometric analysis on LBP related to data analysis has never been performed previously. What is more, we conducted Pearson's correlation analysis with the SPSS software to show development trends in LBP scientifically. Therefore, we could provide more valuable information for researchers to identify hot topics and research frontiers on LBP.

### Research Limitations

The limitations that existed in the study should be taken into consideration in further research. First, the electronic database was limited to Web of Science and might left out some influential articles. Second, we aimed to perform research on the global trend of LBP, but non-English articles were excluded, which may have led to selection bias. Third, the cited frequency of the articles is influenced by IF, author, organization, and time of publication (previously published research should be cited more frequently than the recent research). Therefore, there may be some deviations in reflecting the academic impact of the articles by the number of citations. In addition, the retrieve algorithm of WOS was not based on full text; therefore, a few relevant articles may have been missed and some irrelevant articles may have been included, which may have led to some bias for the visualization of LBP.

## Conclusions

The annual number of publications on LBP has increased sharply in the past two decades, showing that LBP has the potential to be studied precisely and is getting more and more attention. Developed countries like the United States is leading this research field with the largest number of publications; therefore, Chinese researchers should absorb the experience of other countries to identify gaps in the current scientific knowledge that need to be addressed in future research and promote the development of research on LBP. Our study offered insight into the trend of LBP, which may help researchers to explore new directions for future research in this field.

## Data Availability Statement

The original contributions presented in the study are included in the article/supplementary material, further inquiries can be directed to the corresponding author/s.

## Author Contributions

FH and BZ were responsible for concept and design. CW, BZ, SZ, YX, ZL, and CH for drafting the article and its critical revision. ZF and SW for approval of the article. All authors contributed to the article and approved the submitted version.

## Funding

This study was supported by National Natural Science Foundation of China [Grant No: 81874511], Guangdong Provincial Department of Finance Project [Grant (2016) No: 387], Innovation and Entrepreneurship Program for College Students of Guangzhou University of Chinese Medicine (Grant No: 201910572001).

## Conflict of Interest

The authors declare that the research was conducted in the absence of any commercial or financial relationships that could be construed as a potential conflict of interest.

## Publisher's Note

All claims expressed in this article are solely those of the authors and do not necessarily represent those of their affiliated organizations, or those of the publisher, the editors and the reviewers. Any product that may be evaluated in this article, or claim that may be made by its manufacturer, is not guaranteed or endorsed by the publisher.
